# Psychoneurobiology Research and Personalized Treatment of Schizophrenia

**DOI:** 10.3390/jpm11121319

**Published:** 2021-12-07

**Authors:** Tomiki Sumiyoshi

**Affiliations:** Department of Preventive Intervention for Psychiatric Disorders, National Institute of Mental Health, National Center of Neurology and Psychiatry, 4-1-1-Ogawahigashi-cho, Kodaira, Tokyo 187-8551, Japan; sumiyot@ncnp.go.jp

Psychoneurobiological approaches have been used to develop effective treatments for unmet needs in schizophrenia, e.g., some types of cognitive disturbances. To overcome the paucity of breakthrough therapeutics, observations from molecular biology, brain sciences, and their combinations need to be integrated. Besides the development of innovative compounds, there has been a trend towards non-pharmacological therapeutics, including cognitive rehabilitation and neuromodulation (e.g., non-invasive brain stimulation), targeting schizophrenia. These endeavors should bear individualized medicine in mind to optimize the risk/benefit ratios of pharmacotherapy and other modalities of intervention. This Special Issue, consisting of 10 articles, provides a forum for researchers interested in the phenomenology, underlying mechanisms, and treatment of schizophrenia.

Structural and functional anomalies in the brains of individuals with schizophrenia have been identified to promote more effective intervention strategies for the illness. Accordingly, Takahashi et al. [1] observed derangement of temporal lobe structures (e.g., Heschl’s gyrus) in subjects with schizotypal disorder. As similar anatomical findings have been noted in patients with schizophrenia, the finding suggests a common neural substrate across these schizophrenia-spectrum disorders. These lines of morphological changes that are characteristic of schizophrenia are often reflected in neurophysiological observations. Thus, Higuchi et al. [2] examined the P300 component of event-related potentials derived from EEG in people with vulnerability to developing schizophrenia, or in those in an “at-risk mental state (ARMS)”. At baseline, the P300 latencies in ARMS subjects who later converted to schizophrenia were longer compared to those in ARMS non-converters, providing a feasible electrical marker to facilitate early intervention into psychosis.

The search for objective markers to predict a variety of clinical manifestations associated with schizophrenia is enriched by the use of biochemical and genetic findings; for example, Barrera-Conde et al. [3] report the relationship between cannabis use and expressions of RNA-related proteins in olfactory neuroepithelium (ON) cells from clinical samples. This approach is expected to help understand the link between specific proteomic alterations and the increased risk of substance abuse in patients with the illness. Reducing the chance of metabolic syndrome (MetS) caused by antipsychotic drugs has been a major concern from the standpoint of precision medicine. Among several subtypes of serotonin (5-HT) receptors, Paderina et al. [4] found that allelic variants of genes encoding 5HT_2C_ receptors (*HTR2C*) were associated with the incidence of MetS in patients with schizophrenia, highlighting the importance of genetic examinations before starting medications.

It is noteworthy that neurobiological findings in preclinical research have frequently advanced the knowledge in this field, to improve clinical studies. Accordingly, Nitta et al. [5] present animal data on single-nucleotide polymorphisms (SNPs) of genes encoding Piccolo, a presynaptic cytomatrix protein. Specifically, mutant mice with reduced expression of Piccolo in their prefrontal cortex exhibited behavioral and neurochemical abnormalities reminiscent of schizophrenia. The same group of investigators also provide a review of the dysregulated immune system implicated in the pathophysiology of the disease [6]. In particular, the role of the overactivation of synaptic pruning (elimination) by microglia is discussed, suggesting that modulation of the interaction between microglia and synapses may provide an avenue to novel therapeutics.

In addition to psychotic symptoms, some of the key areas of cognitive function, e.g., neurocognition, social cognition, and metacognition, have attracted growing interest to improve social outcomes, or functionality, in schizophrenia patients. Here, Birules et al. [7] examined the time course of the effect of metacognitive training (MCT) on cognitive insight in patients with schizophrenia. The results indicate the benefits of MCT for reducing self-certainty, a component of mega-cognition, which became evident after repeated administrations. On the other hand, Okano et al. [8] report an expert consensus on which assessment tools are to be used to evaluate social cognition in Japanese populations. Considerations were placed on reliability and validity, as well as international comparability and clinical usefulness. This initiative to develop a comprehensive set of social cognition tests is embodied by the Evaluation Study of Social Cognition Measures in Japan (ESCoM), as reported by Kubota et al. [9]. As a novel treatment approach, Yamada et al. [10] are conducting a clinical trial to determine if transcranial direct current stimulation on the temporal lobe sulcus regions alleviates social cognition impairment in schizophrenia.

Overall, the information provided by the authors contributing to this Special Issue will facilitate the development of personal therapeutics of greater clinical value.

## Data Availability

Not applicable.
